# Multilocus Sequence Typing Tool for *Cyclospora cayetanensis*

**DOI:** 10.3201/eid2208.150696

**Published:** 2016-08

**Authors:** Yaqiong Guo, Dawn M. Roellig, Na Li, Kevin Tang, Michael Frace, Ynes Ortega, Michael J. Arrowood, Yaoyu Feng, Yvonne Qvarnstrom, Lin Wang, Delynn M. Moss, Longxian Zhang, Lihua Xiao

**Affiliations:** Centers for Disease Control and Prevention, Atlanta, Georgia, USA (Y. Guo, D.M. Roellig, K. Tang, M. Frace, M.J. Arrowood, Y. Qvarnstrom, D.M. Moss, L. Xiao);; East China University of Science and Technology, Shanghai, China (Y. Guo, N. Li, Y. Feng, L. Wang);; University of Georgia, Griffin, Georgia, USA (Y. Ortega);; Henan Agricultural University, Zhengzhou, China (L. Zhang)

**Keywords:** Cyclospora cayetanensis, whole genome sequencing, multilocus sequence typing, molecular epidemiology, protozoa, parasites

## Abstract

Because the lack of typing tools for *Cyclospora cayetanensis* has hampered outbreak investigations, we sequenced its genome and developed a genotyping tool. We observed 2 to 10 geographically segregated sequence types at each of 5 selected loci. This new tool could be useful for case linkage and infection/contamination source tracking.

*Cyclospora cayetanensis* is an emerging parasitic pathogen responsible for numerous foodborne outbreaks of cyclosporiasis in North America, primarily associated with imported fresh produce from cyclosporiasis-endemic areas (*1*). The lack of genotyping tools has hampered case linkage and infection/contamination source tracking (*2*). In this study, we developed a multilocus sequence typing (MLST) tool to help with identification of this protozoan.

## The Study

To identify potential genotyping markers, we sequenced the genome of 1 *C. cayetanensis* isolate (CHN_HEN01) from Henan, China (*3*), and searched for microsatellite and minisatellite sequences among the first 40 of 4,811 assembled contigs by using Tandem Repeat Finder software (http://tandem.bu.edu/trf/trf.html). We designed primers for nested PCR analysis of the targets based on flanking nucleotide sequences.

The total volume of PCR mixture was 50 μL, which contained 1 μL of DNA (for primary PCR) or 2 μL of the primary PCR product, 250 nmol/L primers, 3 mmol/L magnesium chloride, 200 μmol/L deoxynucleotide triphosphates, 1× GeneAmp PCR buffer (Applied Biosystems, Foster City, CA, USA), and 1.5 U of Taq polymerase (Promega, Madison, WI, USA). The amplification consisted of an initial denaturation at 94°C for 5 min; 35 cycles at 94°C for 45 s; a specified annealing temperature (Table; Technical Appendix Table 1) for 45 s and 72°C for 1 min; and a final extension at 72°C for 7 min. The secondary PCR products were sequenced in both directions on an ABI 3130 Genetic Analyzer (Applied Biosystems).

**Table:** Primer sequences of microsatellite loci used in multilocus sequence analysis of *Cyclospora cayetanensis*

Locus	Contig no.	Targeted repeat*	Primer sequence, 5′ → 3′†	Annealing temp, °C	Expected size, bp	Amplification efficiency, no. positive/no. analyzed
CYC3	00003	TGTA_63_ and TATA_23_	F1: GAAGATGAAGCGTTGGTACG; R1: TACCGCTGCTGGAGTGCAT; F2: TTGTGCATGGCACCCAATGC; R2: CCAGACAGTAGTTCGTGTCTT	55	598	4/4
CYC13	00008	GAT_15_	F1: TTGGAGCAGGACGAGTTTCG; R1: ATGGAAGCGGCTATGAAATTGG; F2: CCTCGGAGTCCTCTGAGTG; R2: AGCCGTCGCAGTGTGTAGCA	58	595	4/4
CYC15	00009	TGC_11_	F1: AGTAGCTACGTGCCAAGACGA; R1: TCGTTCTATCTGACCATAGTAGTG; F2: CGCTGTGCAAGAGGCGATCTA; R2: AAGCACTGCAGGGTCCGTAAC	58	609	4/4
CYC21	00036	AT_31_	F1: TAGTGGCGACTGCGACATG; R1: GCACCTTGCTGATGAGGCA; F2: CTA AGGCTGTCTTGAGCGG; R2: CGCCCACATGCTTCGTATAC	55	471	4/4
CYC22	00037	AC_20_	F1: CACTATGCCGTGTGACACGT; R1: GTAGATTTGCAAGAACTCATGCTA; F2: ATAGTATTCAGGCGCAAACTAAG; R2: GAGGCTTTCCAAAGGTCTAGTT	55	512	4/4

*Tandem repeat identified in the sequence from whole genome sequencing. †Six *C. cayetanensis* specimens were used in initial evaluation of PCR primers: specimens 22231, 22234, 22238, and 28709 were used to evaluate PCR primers from loci CYC3, whereas specimens 22231, 22234, 24550, and 24552 were used for PCR primers from the remaining loci.

The sequences obtained from each locus were aligned by using ClustalX version 2.1 (http://www.clustal.org). A neighbor-joining analysis was used to assess the genetic relatedness of various *C. cayetanensis* sequences for each locus and concatenated sequences of 5 loci. Unique sequences generated from the 5 MLST loci were deposited in GenBank (accession nos. KP723491–KP723518).

Altogether, 15 loci were chosen for evaluations (Table; Technical Appendix Table 1). These loci included 13 microsatellite and 2 minisatellite loci. Six specimens from China and Peru were used in the initial evaluation of the PCR primers designed. Five microsatellite loci (CYC3, CYC13, CYC15, CYC21, and CYC22) exhibiting high PCR amplification efficiency and nucleotide sequence polymorphism in the initial evaluation were chosen for further evaluations of the nature of nucleotide sequence polymorphism by using a total of 64 *C. cayetanensis* specimens from China (n = 26), Nepal (n = 3), Indonesia (n = 1), Guatemala (n = 2), Peru (n = 8), Spain (n = 1), and the United States (n = 23) (Technical Appendix Table 2). Of these, 63 specimens were amplified by PCR at the CYC3 locus, 61 at the CYC13 locus, 63 at the CYC15 locus, 62 at the CYC21 locus, and 64 at the CYC21 locus (Table). However, 1–11 specimens did not produce readable sequences at each locus.

**Figure 1:**
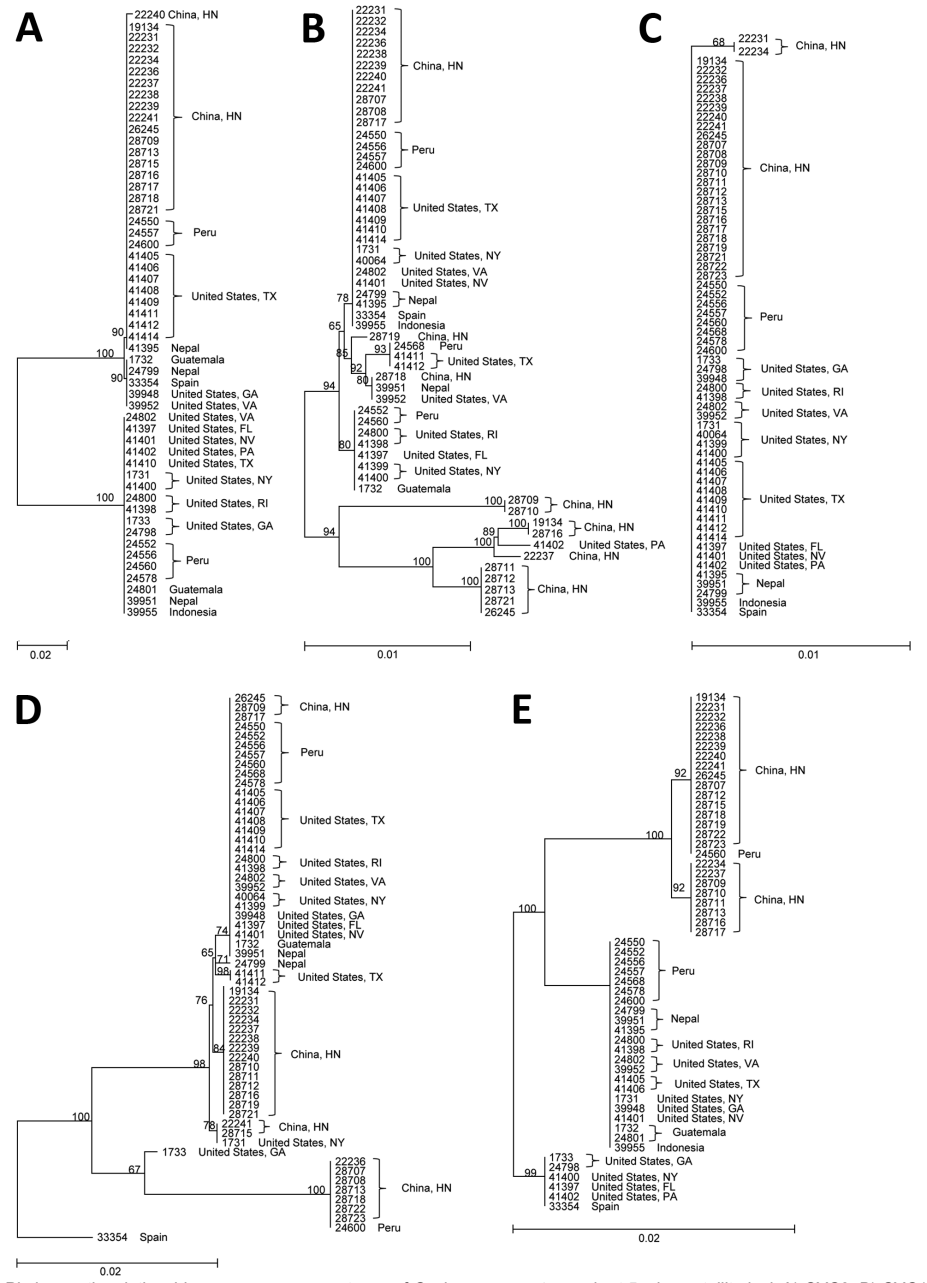
Phylogenetic relationships among sequence types of *Cyclospora cayetanensis* at 5 microsatellite loci: A) CYC3, B) CYC13, C) CYC15, D) CYC21, and E) CYC22. Tree was constructed on the basis of neighbor-joining analyses of the nucleotide sequences, using genetic distances calculated by the Kimura 2-parameter model. Numbers on branches are bootstrap values from 1,000 replicate analyses. Only values >50% are displayed on the left of each node. Scale bars indicate substitution rates per nucleotide. HN, Henan.

Nucleotide sequence alignment led to the identification of 4 sequence types at locus CYC3, 10 at locus CYC13, 2 at locus CYC15, 8 at locus CYC21, and 4 at locus CYC22 (Technical Appendix Table 2). As expected, all 5 loci showed differences in the number of microsatellite repeats. In addition, single nucleotide polymorphisms were present at all loci (Technical Appendix Figures 1–5). Sequences from CYC3, CYC13, CYC21, and CYC22 formed 2–3 major groups in neighbor-joining trees (Figure 1). Clear geographic clustering of sequences was observed at most loci, with specimens from China largely clustering together and US outbreak specimens often clustering with specimens from Peru (Figure 1). Of the 9 specimens from a 2013 Texas outbreak, 1 had a different sequence from the remaining specimens at CYC3, 2 had different sequences from the remaining specimens at CYC13 and CYC21, and at CYC22, PCR products from 7 specimens produced unreadable sequences (Technical Appendix Table 2).

**Figure 2:**
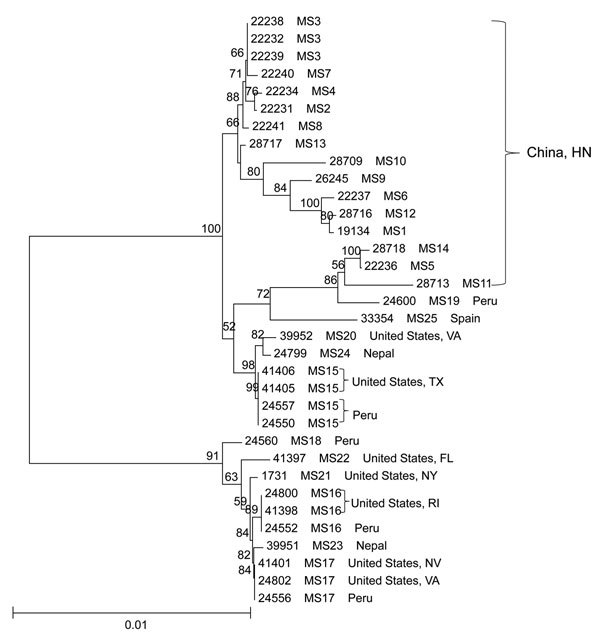
Phylogenetic relationships among concatenated multilocus sequence types of *Cyclospora cayetanensis* as assessed by a neighbor-joining analysis of the nucleotide sequences, using genetic distances calculated by the Kimura 2-parameter model. Numbers on branches are bootstrap values from 1,000 replicate analyses. Only values >50% are displayed on the left of each node. Scale bar indicates substitution rates per nucleotide. HN, Henan.

A total of 34 specimens had complete sequence data at 5 loci, forming 25 MLST types (Technical Appendix Table 2). Most of the MLST types had only 1 specimen, except for 4 MLST types (MS3, MS15, MS16, and MS17), which had 3 or 4 specimens (Technical Appendix Table 2). A neighbor-joining analysis of the concatenated sequences of 2,317 bp showed clear geographic clustering of MLST types (Figure 2). Most specimens from China clustered together in 1 major group, whereas specimens from outbreaks in the United States formed 2 other groups with specimens from Peru. The specimen from Spain appeared to be distinct.

## Conclusions

In this study, we sequenced the genome of *C. cayetanensis* protozoa and developed a genotyping tool. Noticeable geographic clustering was observed at some of the loci, with specimens from China forming 1–2 groups at each of these loci. In contrast, the US outbreak specimens mostly grouped together with Peru specimens, probably because of the imported nature of pathogens from Central and South America. The geographic clustering pattern of specimens from the same country at 1 locus does not conform to patterns at other loci, probably because of the occurrence of genetic recombination among parasites in a particular area. Therefore, the use of a single genetic marker is probably not useful in geographic tracking of infection sources of this species.

Data generated from this study have demonstrated the high resolution of the MLST tool. Although genotyping resulted in complete data at all 5 loci for only 34 of the 64 specimens, 25 MLST types were detected. The failure in obtaining informative sequences from some amplicons was mainly attributable to the presence of PCR products with different repeat lengths, leading to overlapped signals following the tandem repeat region. This highlights some potential challenges in investigations of cyclosporiasis outbreaks using genotyping tools. Only 2 of the 9 specimens from the 2013 outbreak of cyclosporiasis in Texas produced complete MLST data because of inability to obtain readable sequences from CYC22. Sequence analysis at other loci suggested that at least 3 types of *C. cayetanensis* protozoa were present in specimens from the outbreak. The occurrence of mixed *C. cayetanensis* populations probably led to unreadable sequences for most specimens from the outbreak at CYC22.

The occurrence of mixed *C. cayetanensis* populations in large outbreaks is expected because divergent MLST types are apparently present in a small community or geographic area. For example, the Peru specimens in this study were from a small shantytown, Pampas de San Juan de Miraflores, in Lima (*4*), but the specimens had at least 5 MLST types among them. Similarly, 14 MLST types were detected among the 26 Chinese specimens collected from 2 neighboring cities (Kaifeng and Zhengzhou) in Henan Province (*5*). Fresh produce is frequently contaminated by *C. cayetanensis* protozoa through irrigation water (*6*) and thus has a higher probability of containing multiple *C. cayetanensis* genotypes. It might be possible to use only 2 or 3 loci that are highly polymorphic and easier to sequence in *C. cayetanensis* genotyping, such as CYC13 and CYC21.

In summary, whole-genome sequence data from *C. cayetanensis* protozoa enabled the development of a MLST tool for characterizing isolates in outbreak investigations. The high resolution of the typing tool and the apparent presence of geographic clusters might facilitate the identification of outbreaks and infection sources. Nevertheless, extensive characterization of specimens from diverse areas and wide application of the developed tool in outbreak investigations are needed to better understand *C. cayetanensis* transmission.

Technical Appendix Primer sequences of additional microsatellite and minisatellite loci selected for initial analysis of *Cyclospora cayetanensis*. Specimens used in the study and their sequence identity at the 5 selected loci. Variations in nucleotide sequences among specimens at the 5 selected loci.
