# Supplementary material for: Multilocus Sequence Typing Tool for Cyclospora cayetanensis
Source: Emerg Infect Dis. 2016 Aug;22(8):1464–7. doi: 10.3201/eid2208.150696 (PMC4982147; doi:10.3201/eid2208.150696)
Supplement: Technical Appendix — Primer sequences of additional microsatellite and minisatellite loci selected for initial analysis of Cyclospora cayetanensis. Specimens used in the study and their sequence identity at the 5 selected loci. Variations in nucleotide sequences among specimens at the 5 selected loci. [file 15-0696-Techapp-s1.pdf]

Article DOI: http://dx.doi.org/10.3201/eid2208.150696

Multilocus Sequence Typing Tool for *Cyclospora cayetanensis*

Technical Appendix

Technical Appendix Table 1. Primer sequences of additional microsatellite and minisatellite loci selected for initial analysis of *Cyclospora cayetanensis*

Locus	Contig no.	Targeted repeat*	Primer sequence, (5′→3′)†	Annealing temp, °C	Expected size, bp	Amplification efficiency, no. positive/no. analyzed
CYC7	00007	AC_{40}	F1: CGATCGTATGGAGAGCTCCT; R1: AAGATGCCCTTCAGGCGGA; F2: TTCACAATGTACGACTTGGCAC; R2: GCGCATCATATACAGATGGTC	55	593	4/4
CYC8	00008	$ACCACC_{16}$	F1: ATTCCACAGTCAAGACTGCG; R1: CTGAGAGATGGTTTCTCTCCA; F2: CTTCCTCGAGGGTATCTTCCAT; R2: GCGCAGAGCTCTCTGCAGA	55	435	4/4
CYC10	00010	$TGTG_{62}$	F1: GTCGATTTCACAGTAGAGGGAT; R1: CTGCTTCGTGGCGAGCAAT; F2: ATGCATGCGTGTTTAGGGCTT; R2: ACAGCGAAATCATTTGCTGAGG	55	690	4/4
CYC12	00012	$ACTTCTTCTCCTTCTGTTCT_{30}$	F1: GAAGGTGCTACGGTGACAACT; R1: TTGAAGCCTCGGATGAGACTT; F2: CGGAGGCGCCAAAGTTGTCT; R2: CTCCACTGTTCCAGCACC	55	701	4/4
CYC14	00016	TG_{22}	F1: TTGGTGTGTCGTCCCTACTAGA; R1: ATACAAAGACGACTAGCATGGC; F2: CCATTCTGCCATCTAAGCATTAA; R2: AACGCATACTTCTGATTGAGGCAT	55	341	4/4
CYC16	00018	TGC_{12}	F1: CACATAAAGAAGCACTCAAGACG; R1: GGTCTCGCATTTAGACACTCG; F2: CAACTCAGGACCCTTGTCGA; R2: CCTCGTAGTAAAAGACCCTTGT	58	553	4/4
CYC17	00020	AGC_{12}	F1: AGCATTGAGCATCCCTCCTG; R1: TCCGCTTCCCATGCAGCTTC; F2: CCTACAGTGTCAGCCGCTTC; R2: ACAGTTGCATGCACATCAGTTC	58	623	4/4
CYC18	00022	$GTGTTTGTTT_{45}$	F1: GCATGCAACTTCTGGTGCATC; R1: AAGTGATTGCAGCAGCGAGGT; F2: TGGAAGGGATGCTCGCTGC; R2: CCCAAGTGCTGCAGCAATAA	55	947	4/4
CYC19	00034	AG_{25}	F1: TCTTAGAGCTCCCTAGTCGG; R1: CTCGTAGGCATCGACTGGAA; F2:	55	361	4/4

Locus	Contig no.	Targeted repeat*	Primer sequence, (5′→3′)†	Annealing temp, °C	Expected size, bp	Amplification efficiency, no. positive/no. analyzed
CYC20	00035	CA_{13}	CGCACGCTGCTGGGTATG; R2: TCTTTCTAAGCGCATGCTGTTAA F1: CTTGCTTCGATTTCCCTACGG; R1: CTGCAGGTTTACGAGCCTG; F2: CATCGTCAGTCACTCGGAGTT; R2: GATGGCTCGGCAACAAGTG	55	421	4/4

*Tandem repeat identified in the sequence from whole genome sequencing.

†Six *C. cayetanensis* specimens were used in initial evaluation of PCR primers: specimens 22231, 22234, 22238, and 28709 were used to evaluate PCR primers from CYC7, CYC8, CYC10, and CYC12, whereas specimens 22231, 22234, 24550, and 24552 were used for the primers from the remaining loci.

Technical Appendix Table 2. Specimens used in the study and their sequence identity at the 5 selected loci

Specimen	Host	Study	Sequence type*					MLST type
			CYC3	CYC13	CYC15	CYC21	CYC22	
19134	Human	China, Henan, 2006	C1	C5	C1	C1	C3	MS1
22231	Human	China, Henan, 2007	C1	C1	C2	C1	C3	MS2
22232	Human	China, Henan, 2007	C1	C1	C1	C1	C3	MS3
22234	Human	China, Henan, 2007	C1	C1	C2	C1	C4	MS4
22236	Human	China, Henan, 2007	C1	C1	C1	C4	C3	MS5
22237	Human	China, Henan, 2007	C1	C6	C1	C1	C4	MS6
22238	Human	China, Henan, 2007	C1	C1	C1	C1	C3	MS3
22239	Human	China, Henan, 2007	C1	C1	C1	C1	C3	MS3
22240	Human	China, Henan, 2007	C4	C1	C1	C1	C3	MS7
22241	Human	China, Henan, 2007	C1	C1	C1	C3	C3	MS8
26245	Human	China, Henan, 2008	C1	C7	C1	C2	C3	MS9
28707	Human	China, Henan, 2009	Noisy	C1	C1	C4	C3	
28708	Human	China, Henan, 2009	Noisy	C1	C1	C4	Noisy	
28709	Human	China, Henan, 2009	C1	C2	C1	C2	C4	MS10
28710	Human	China, Henan, 2009	Noisy	C2	C1	C1	C4	
28711	Human	China, Henan, 2009	Noisy	C7	C1	C1	C4	
28712	Human	China, Henan, 2009	Noisy	C7	C1	C1	C3	
28713	Human	China, Henan, 2009	C1	C7	C1	C4	C4	MS11
28715	Human	China, Henan, 2009	C1	Noisy	C1	C3	C3	
28716	Human	China, Henan, 2009	C1	C5	C1	C1	C4	MS12
28717	Human	China, Henan, 2009	C1	C1	C1	C2	C4	MS13

Specimen	Host	Study	Sequence type* CYC3	CYC13	CYC15	CYC21	CYC22	MLST type
28718	Human	China, Henan, 2009	C1	C4	C1	C4	C3	MS14
28719	Human	China, Henan, 2009	Noisy	C8	C1	C1	C3	
28721	Human	China, Henan, 2009	C1	C7	C1	C1	Noisy	
28722	Human	China, Henan, 2009	Noisy	Noisy	C1	C4	C3	
28723	Human	China, Henan, 2009	Noisy	Noisy	C1	C4	C3	
24550	Human	Peru, 2006	C1	C1	C1	C2	C1	MS15
24552	Human	Peru, 2006	C3	C3	C1	C2	C1	MS16
24556	Human	Peru, 2006	C3	C1	C1	C2	C1	MS17
24557	Human	Peru, 2006	C1	C1	C1	C2	C1	MS15
24560	Human	Peru, 2006	C3	C3	C1	C2	C3	MS18
24568	Human	Peru, 2006	–	C9	C1	C2	C1	
24578	Human	Peru, 2006	C3	Noisy	C1	C2	C1	
24600	Human	Peru, 2006	C1	C1	C1	C4	C1	MS19
1733	Human	United States, Georgia, 1996	C3	–	C1	C8	C2	
24798	Human	United States, Georgia, 1996	C3	–	C1	–	C2	
39948	Human	United States, Georgia, 1996	C2	Noisy	C1	C2	C1	
24800	Human	United States, Rhode Island, 1997	C3	C3	C1	C2	C1	MS16
41398	Human	United States, Rhode Island, 1997	C3	C3	C1	C2	C1	MS16
24802	Human	United States, Virginia, 1998	C3	C1	C1	C2	C1	MS17
39952	Human	United States, Virginia, 1998	C2	C4	C1	C2	C1	MS20
1731	Human	United States, New York, 1998	C3	C1	C1	C3	C1	MS21
40064	Human	United States, New York, 1998	Noisy	C1	C1	C2	Noisy	
41399	Human	United States, New York, 1997	Noisy	C3	C1	C2	Noisy	
41400	Human	United States, New York, 1997	C3	C3	C1	Noisy	C2	
41405	Human	United States, Texas, 2013	C1	C1	C1	C2	C1	MS15
41406	Human	United States, Texas, 2013	C1	C1	C1	C2	C1	MS15
41407	Human	United States, Texas, 2013	C1	C1	C1	C2	Noisy	
41408	Human	United States, Texas, 2013	C1	C1	C1	C2	Noisy	
41409	Human	United States, Texas, 2013	C1	C1	C1	C2	Noisy	
41410	Human	United States, Texas, 2013	C3	C1	C1	C2	Noisy	
41411	Human	United States, Texas, 2013	C1	C9	C1	C5	Noisy	
41412	Human	United States, Texas, 2013	C1	C9	C1	C5	Noisy	

Specimen	Host	Study	Sequence type*					MLST type
			CYC3	CYC13	CYC15	CYC21	CYC22	
41414	Human	United States, Texas, 2013	C1	C1	C1	C2	Noisy	
41397	Human	United States, Florida, 1996	C3	C3	C1	C2	C2	MS22
41401	Human	United States, Nevada, 1997	C3	C1	C1	C2	C1	MS17
41402	Human	United States, Pennsylvania, 2000	C3	C10	C1	Noisy	C2	
41395	Human	Nepal, 1997	C1	C1	C1	Noisy	C1	
39951	Human	Nepal, 1997	C3	C4	C1	C2	C1	MS23
24799	Human	Nepal, 1997	C2	C1	C1	C6	C1	MS24
39955	Human	Indonesia, 2012	C3	C1	C1	Noisy	C1	
33354	Human	Spain, 2011	C2	C1	C1	C7	C2	MS25
24801	Human	Guatemala, 1997	C3	–	–	–	C1	
1732	Human	Guatemala, 1997	C2	C3	Noisy	C2	C1	

*Dashes indicate no PCR amplification; “noisy” represents unreadable sequence. MLST, multilocus sequence typing.

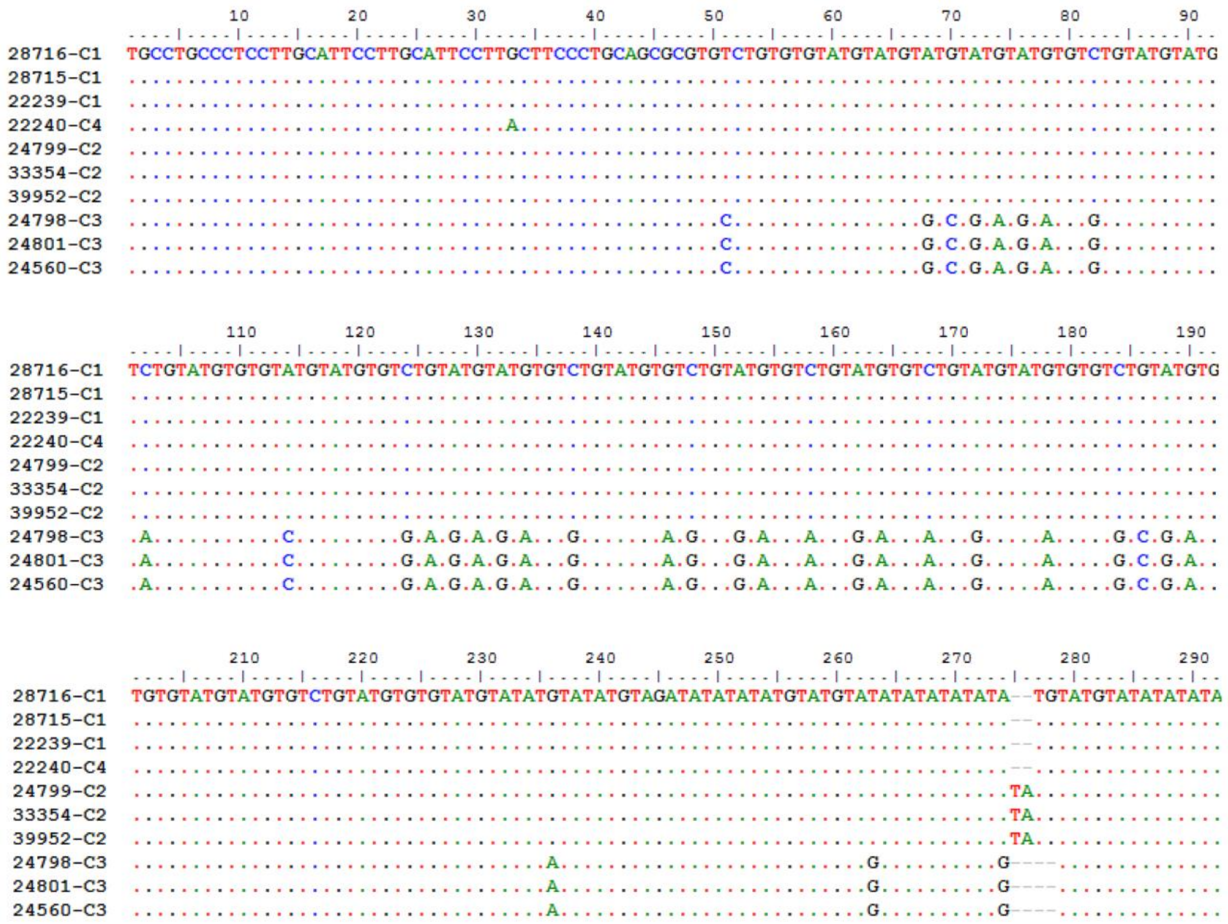

Technical Appendix Figure 1. Variations in nucleotide sequences among *Cyclospora cayetanensis* specimens at the CYC3 locus. Dots denote nucleotides identical to those in the first sequence of the sequence alignment, and dashes denote nucleotide deletions.

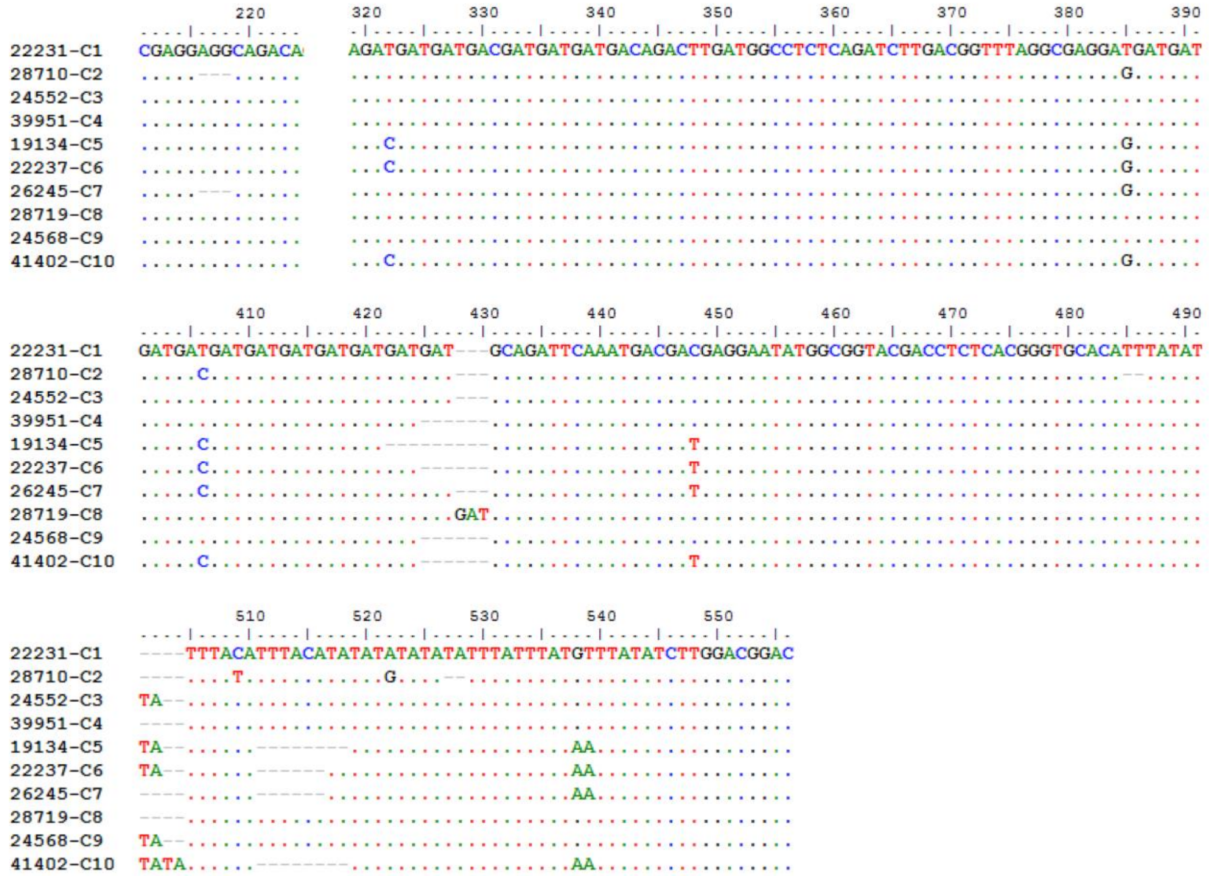

Technical Appendix Figure 2. Variations in nucleotide sequences among *Cyclospora cayetanensis* specimens at the CYC13 locus. Dots denote nucleotides identical to those in the first sequence of the sequence alignment, and dashes denote nucleotide deletions.

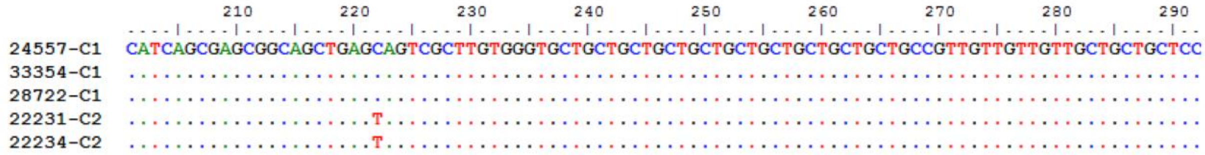

Technical Appendix Figure 3. Variations in nucleotide sequences among *Cyclospora cayetanensis* specimens at the CYC15 locus. Dots denote nucleotides identical to those in the first sequence of the sequence alignment, and dashes denote nucleotide deletions.

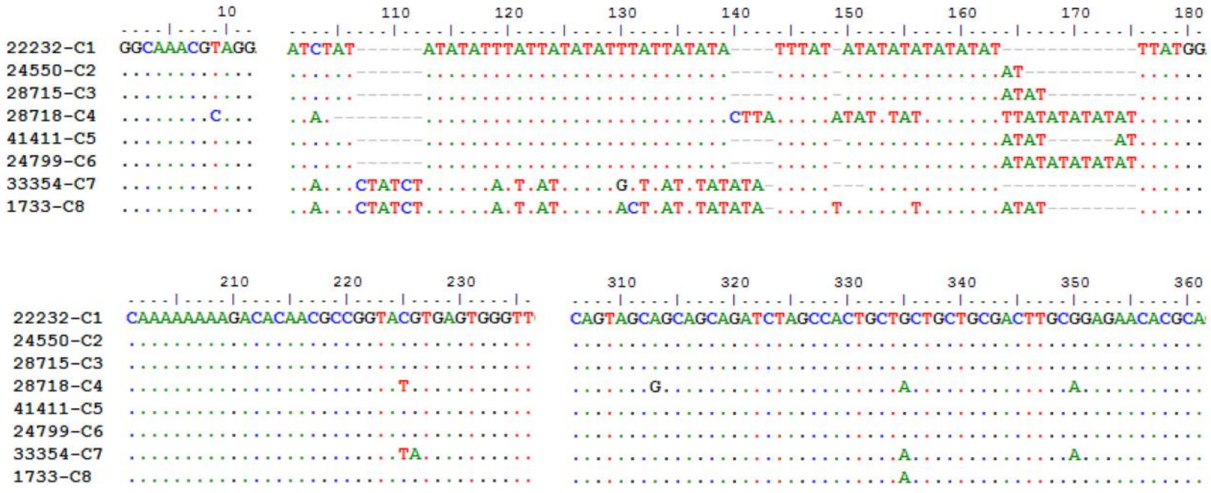

Technical Appendix Figure 4. Variations in nucleotide sequences among *Cyclospora cayetanensis* specimens at the CYC21 locus. Dots denote nucleotides identical to those in the first sequence of the sequence alignment, and dashes denote nucleotide deletions.

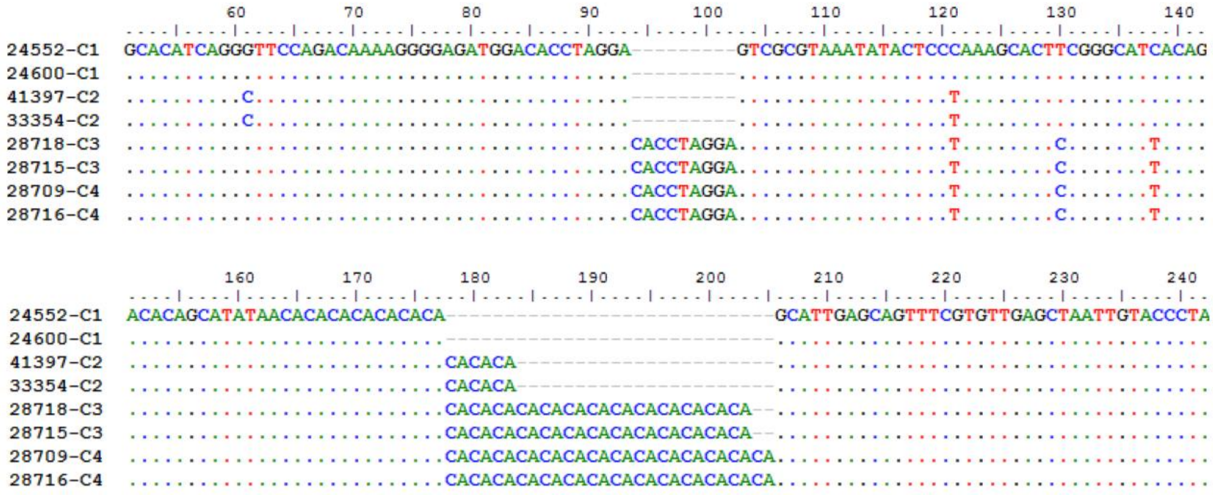

Technical Appendix Figure 5. Variations in nucleotide sequences among *Cyclospora cayetanensis* specimens at the CYC22 locus. Dots denote nucleotides identical to those in the first sequence of the sequence alignment, and dashes denote nucleotide deletions.
